# Comprehensive Evaluation of *Vacrol Oil Combination* in Experimental Wound Healing: From Phytochemical Analysis to Functional and Structural Repair

**DOI:** 10.3390/plants14223547

**Published:** 2025-11-20

**Authors:** Esra Küpeli Akkol, Didem Deliorman Orhan

**Affiliations:** Department of Pharmacognosy, Faculty of Pharmacy, Gazi University, Etiler, Ankara 06330, Türkiye; didem@gazi.edu.tr

**Keywords:** vacrol oil combination, wound healing, essential oils, carvacrol, hydroxyproline, hyaluronidase inhibition, histopathology, phytotherapeutics

## Abstract

Wound healing is a complex biological process involving overlapping phases of inflammation, proliferation, and remodeling. Plant-derived agents have gained attention as alternatives or adjuncts to synthetic drugs owing to their accessibility and favorable safety profile. This study evaluated the wound-healing activity of Vacrol Oil Combination (*VOC*), a phytotherapeutic preparation, through in vivo wound models and in vitro enzyme inhibition assays. Linear incision wounds in rats and circular excision wounds in mice were treated with *VOC*, administered orally, topically, or in combination for 10 days. Experimental groups included a negative control (no treatment), a vehicle control (olive oil), *VOC*-treated groups, and a reference group treated with 0.2% nitrofurazone. Wound contraction, tensile strength, histopathology, and hydroxyproline levels were assessed. In vitro assays were conducted to evaluate the inhibitory effects of *VOC* on hyaluronidase, collagenase, and elastase. VEGF and TGF-β1 levels were measured to assess the involvement of growth factors in the healing process. The chemical composition of *VOC* was characterized by gas chromatography–mass spectrometry (GC–MS), which identified carvacrol as the major compound, together with 1,8-cineole, linalool, eugenol, and cinnamaldehyde as prominent constituents known for their anti-inflammatory and antioxidant activities. *VOC* treatment significantly enhanced wound contraction and tensile strength compared to controls, with the oral + topical group showing the highest efficacy. Hydroxyproline levels and histological findings confirmed improved collagen synthesis and tissue regeneration. GC–MS analysis identified carvacrol as the major constituent of *VOC*, along with eugenol and linalool, which are known for their anti-inflammatory and antioxidant effects. Furthermore, *VOC* increased tissue levels of VEGF and TGF-β1, suggesting a role in stimulating angiogenesis and extracellular matrix remodeling. These findings indicate that the phytoconstituents of *VOC*, particularly carvacrol and oxygenated terpenes, act synergistically to promote wound repair. *VOC* demonstrates strong potential as a complementary phytotherapeutic agent for wound management, warranting further clinical investigation.

## 1. Introduction

Wound healing is a dynamic and complex biological process comprising overlapping phases of hemostasis, inflammation, proliferation, and remodeling [1]. Disturbances or delays in these tightly regulated events may result in chronic wounds, which remain a major clinical challenge due to their high prevalence, prolonged healing time, and considerable socioeconomic burden [2]. The extracellular matrix (ECM), largely composed of collagen, elastin, and glycosaminoglycans, plays a central role in tissue repair. Enzymes such as hyaluronidase, collagenase, and elastase regulate ECM remodeling; however, excessive enzymatic activity leads to the degradation of structural proteins, impairs healing, and contributes to the pathophysiology of chronic wounds [3,4].

Conventional wound management relies on topical antimicrobials, antibiotics, and synthetic dressings. Although these strategies may reduce infection and promote healing, their clinical application is limited by adverse effects, high cost, and the increasing threat of antimicrobial resistance [5]. In this context, natural products and phytochemicals have gained renewed attention as safer, accessible, and cost-effective alternatives. Various plant-derived compounds, particularly polyphenols and terpenoids, have shown potent anti-inflammatory, antioxidant, and protease-inhibitory properties that facilitate tissue repair [6,7,8].

Essential oils are complex mixtures of volatile terpenoids, phenolics, and aromatic compounds exhibiting broad pharmacological actions, including antimicrobial, antioxidant, and wound-healing effects [9,10,11]. Their biological activities are primarily attributed to major constituents such as carvacrol, thymol, linalool, cineole, and eugenol, which have been reported to enhance fibroblast proliferation, angiogenesis, and collagen synthesis [12,13,14]. However, most previous studies have examined individual essential oils, while the therapeutic potential of synergistic essential oil combinations remains underexplored.

Vacrol Oil Combination (*VOC*) is a standardized phytotherapeutic formulation enriched with essential oils derived from *Origanum onites* L., *Eucalyptus globulus* Labill., *Rosmarinus officinalis* L., *Salvia officinalis* L., and *Juniperus communis* L. This combination provides controlled dosing, improved bioavailability, and the potential for synergistic wound-healing effects. Preliminary GC–MS analysis confirmed the presence of bioactive constituents such as carvacrol, 1,8-cineole, linalool, and eugenol, all known for their anti-inflammatory, antimicrobial, and tissue-regenerative properties.

Given the increasing demand for evidence-based natural therapeutics, the systematic evaluation of *VOC* is warranted. Preliminary experimental findings suggest that certain components of *VOC* may promote wound healing through several mechanisms, including the stimulation of collagen deposition, the acceleration of epithelialization, and the modulation of proteolytic enzyme activity. Despite growing evidence supporting the wound-healing efficacy of individual essential oils, comprehensive scientific validation of multi-component formulations like *VOC* in standardized in vivo wound models remains limited. Therefore, the present study was designed to (i) characterize the chemical composition of *VOC* using GC–MS, and (ii) investigate its wound-healing potential through established in vivo incision and excision wound models, supported by histopathological assessment, hydroxyproline quantification, and in vitro enzyme inhibition assays. These approaches aimed to provide an integrative understanding of the role of *VOC* in promoting wound repair.

## 2. Results

### 2.1. GC–MS Profile Chromatographic Pattern

*VOC* exhibited a complex phytochemical composition with 28 identified compounds. The major constituent was carvacrol (50.1 ± 0.1% *w*/*w*), followed by 1,8-cineole (9.7 ± 0.1% *w*/*w*), linalool (6.3 ± 0.1% *w*/*w*), eugenol (4.7 ± 0.1% *w*/*w*), and cinnamaldehyde (4.3 ± 0.1% *w*/*w*). Minor constituents included α-pinene (3.5%), *p*-cymene (3.2%), thymol (2.1%), menthol (1.9%), and bisabolene (1.1%). A complete list of compounds with their retention times and concentrations is provided in Table 1.

**Figure 1:**
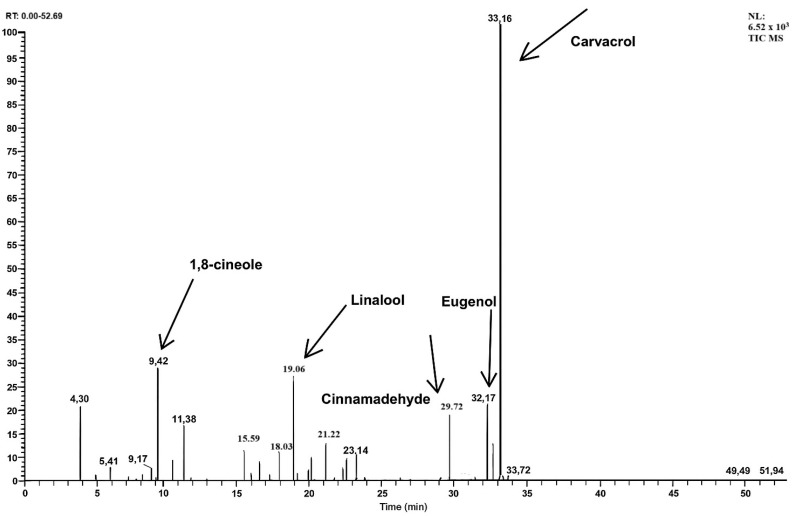
GC-MS chromatogram of Vacrol Oil Combination.

The GC–MS chromatogram (Figure 1) confirmed distinct peaks corresponding to carvacrol as the predominant compound, along with other terpenes and phenolic constituents.

Comparison with reported compositions of the individual essential oils forming the *VOC*—namely *O. onites* (oregano), *E. globulus* (eucalyptus), *R. officinalis* (rosemary), *S. officinalis* (sage), and *J. communis* (juniper)—revealed both shared and distinctive chemical features. Consistent with oregano oil, *VOC* contained carvacrol as the dominant component, supported by smaller amounts of thymol and *p*-cymene. However, *VOC* also incorporated abundant oxygenated terpenes such as 1,8-cineole, linalool, and eugenol, derived from eucalyptus, rosemary, and sage oils, respectively. These constituents are rarely present together in single-source essential oils. The combination of phenolic monoterpenes with oxygenated terpenes and aromatic compounds confers a broader pharmacological potential—particularly antioxidant and anti-inflammatory activity—highlighting the synergistic and unique chemical profile of the *VOC*.

### 2.2. Linear Incision Wound Model

The linear incision wound model in rats was used to assess whether *VOC* contributes to wound healing by enhancing epithelial proliferation and underlying tissue remodeling. After 10 days of treatment, wound tensile strength was measured. Topical *VOC* administration increased tensile strength by 46.4%, whereas combined oral and topical administration enhanced tensile strength by 74.8% compared with the negative control (Table 2). The tensile strength in the combined treatment group was comparable to that of the reference drug, nitrofurazone.

**Figure 2:**
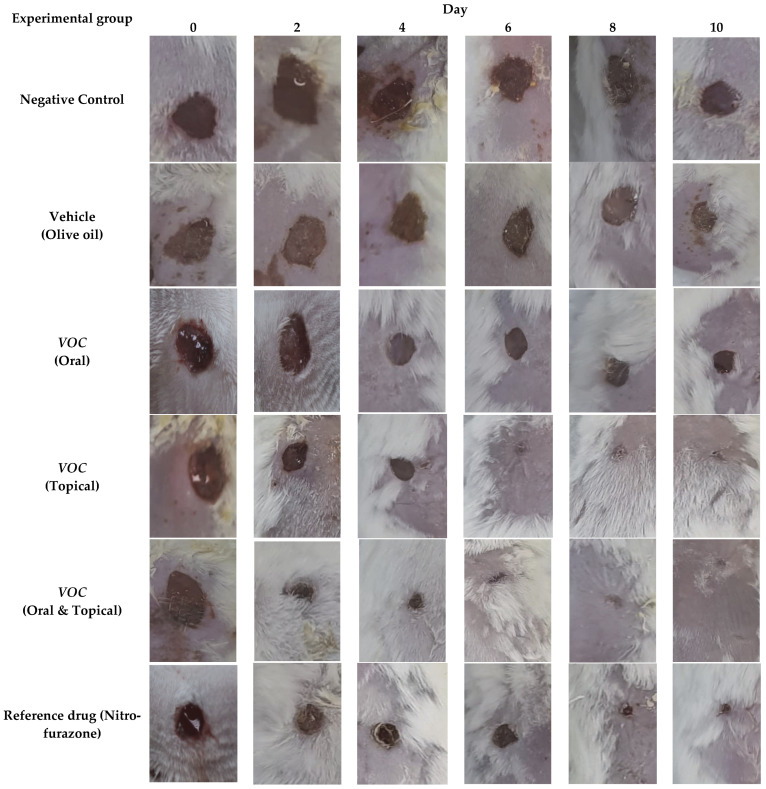
Representative photographs of excision wounds in different treatment groups were captured on alternate days during the 10-day experimental period. The serial images illustrate the progressive wound closure patterns under different treatment conditions. High-resolution representative images are provided for improved visualization of wound contraction.

Representative photographs of excision wounds in different treatment groups are presented in Figure 2. The serial images, captured on alternate days over a 10-day experimental period, illustrate the progressive wound closure patterns under different treatment conditions. Notably, the treated groups exhibited accelerated wound contraction compared to the control groups, highlighting the therapeutic efficacy of the interventions (Figure 2).

### 2.3. Circular Excision Wound Model

In the circular excision wound model, wound contraction was evaluated by measuring wound area reduction over a 10-day treatment period. On day 10, topical *VOC* achieved 63.5% contraction, while combined oral and topical *VOC* treatment resulted in 100% wound contraction (Table 3). In contrast, nitrofurazone-treated tissues showed 80.5% healing on day 10, whereas the vehicle control (olive oil) achieved only 28.4% contraction.

### 2.4. Histopathological Findings

**Figure 3:**
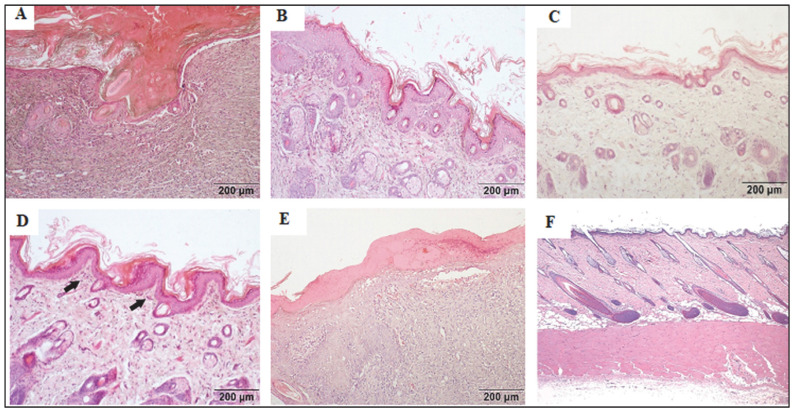
Representative histological sections of wound tissues stained with hematoxylin–eosin (H&E). (**A**) Negative control showing severe inflammation, necrosis, and hyperkeratosis. (**B**) Vehicle control (olive oil) with persistent inflammation and necrosis. (**C**) Nitrofurazone-treated tissue showing organized collagen bundles and re-epithelialization. (**D**) Oral *VOC* showing mild hyperkeratosis and moderate inflammation. (**E**) Topical *VOC* showing mild hyperkeratosis with minimal inflammation. (**F**) Combined oral + topical *VOC* demonstrating nearly complete re-epithelialization and remodeling.

Histopathological evaluation revealed remodeling and re-epithelialization in tissues treated with nitrofurazone and combined oral + topical *VOC*. In the negative control and vehicle control groups, marked inflammation, necrosis, and hyperkeratosis were observed. Oral *VOC* treatment resulted in mild hyperkeratosis with moderate inflammation, while topical *VOC* led to mild hyperkeratosis with only mild inflammation. Representative histological images stained with hematoxylin–eosin (H&E) is shown in Figure 3.

### 2.5. Hydroxyproline Content

Collagen, an essential ECM protein, is critical for rapid wound healing. Hydroxyproline levels, used as an indirect marker of collagen content (µg/mg tissue), were measured in wound tissues. Treatment with *VOC* significantly increased hydroxyproline levels compared with control. Both topical *VOC* and combined oral + topical *VOC* groups showed marked elevations in hydroxyproline content, indicating enhanced collagen deposition (Table 4).

### 2.6. In Vitro Enzyme Inhibition Assays

Hyaluronidase is a glycosidase that depolymerizes hyaluronan, collagenase from *C. histolyticum* is a metalloproteinase, and human neutrophil elastase is a serine protease. Excessive activity of these enzymes contributes to ECM degradation and delayed healing. *VOC* demonstrated potent inhibition of hyaluronidase, with an inhibition rate of 71.63% at 100 µg/mL. However, *VOC* showed no significant inhibitory effects against collagenase or elastase (Table 5).

### 2.7. Effect of VOC on the Healing Growth Factors (VEGF and TGF-β1) of Wound Tissue

**Figure 4:**
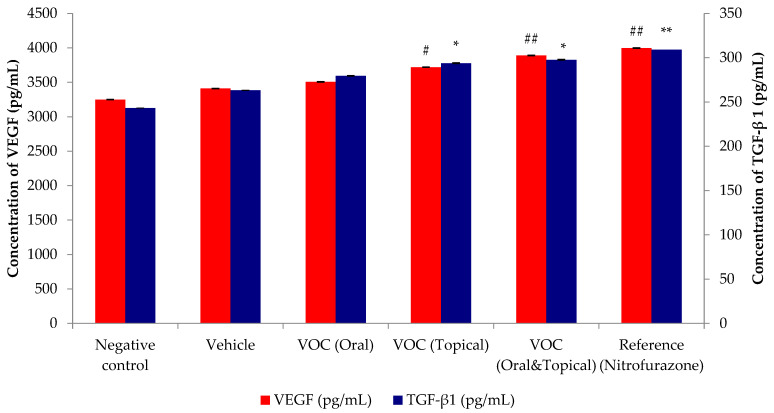
Secretions of growth factors like VEGF and TGF-β1 on day 10 with treatment of test materials. All values are expressed as mean ± SEM. Each group consists of seven mice. #: *p*  < 0.05 and ##: *p*  < 0.01 test group compared to negative control group; *: *p* < 0.05 and **: *p* < 0.01 treatments groups compared to negative control group.

Figure 4 illustrates the alterations in mouse growth factor levels that occur throughout this study. In general, an increase in growth factor production was observed in response to treatment with *VOC* (oral and topical) 10 days after the induction of injury. The wound tissues from the negative control group exhibited levels of 3257 ± 242 pg/mL VEGF and 254 ± 27 pg/mL TGF-β1 on day 10. Treatment with *VOC* (oral and topical) led to a significant enhancement in the synthesis of VEGF (3892 ± 205 pg/mL) and TGF-β1 (311 ± 19 pg/mL) in wound samples when compared to other groups. In a similar manner, the reference drug (nitrofurazone) that was employed demonstrated a substantial increase in both growth factors in wounded tissues (Figure 4).

## 3. Discussion

The GC–MS analysis revealed that carvacrol constitutes more than half of the *VOC*’s composition, making it the principal bioactive component. Carvacrol is a monoterpenoid phenol with well-documented antimicrobial, antioxidant, and wound-healing activities [9]. Its high abundance strongly suggests that it contributes significantly to the observed pharmacological effects. Beyond carvacrol’s predominance, systematic evidence indicates that carvacrol/thymol support multiple stages of wound repair, including inflammation control, re-epithelialization, granulation, and collagen deposition, and carvacrol can enhance angiogenic paracrine signaling in mesenchymal cells [10,11]. Alongside carvacrol, *VOC* contains 1,8-cineole and linalool, both of which are known for their anti-inflammatory and analgesic properties [12,13]. Eucalyptol (1,8-cineole) exhibits clinically relevant anti-inflammatory actions and accelerates wound closure when applied topically, while linalool provides additional analgesic and anti-inflammatory support [12,13,14]. These constituents may act synergistically to enhance tissue repair and reduce inflammatory responses at the wound site. Furthermore, eugenol and cinnamaldehyde, identified in meaningful amounts, possess strong antimicrobial and antioxidant properties [15,16,17], potentially protecting wounds against microbial colonization while reducing oxidative stress. Given the documented synergistic effects among essential oil constituents, *VOC*’s multi-terpene profile plausibly yields additive or synergistic antimicrobial effects [18].

Other minor constituents, such as *p*-cymene, thymol, menthol, and bisabolene, broaden the phytochemical profile of the *VOC*, and are reported to exert complementary effects such as modulation of inflammation, enhanced angiogenesis, and antimicrobial action [18]. Collectively, this diverse phytochemical spectrum suggests that *VOC*’s wound-healing activity results from the synergistic interplay of multiple compounds rather than a single active ingredient.

Functionally, *VOC* treatment significantly improved wound repair outcomes. In the incision model, combined oral and topical administration enhanced tensile strength to levels comparable to the reference wound-healing agent nitrofurazone. This effect may be attributed to enhanced collagen cross-linking and stabilization, consistent with previous findings that natural products can improve dermal integrity Via ECM modulation [19,20]. Similarly, in the excision model, complete wound contraction observed by day 10 in the combined treatment group highlights *VOC*’s strong epithelialization and proliferative-promoting capacity, aligning with earlier studies showing botanical preparations can accelerate wound closure [21].

Histopathological findings further supported these outcomes. *VOC* treatment reduced inflammation and necrosis while enhancing re-epithelialization and remodeling. The nearly normal tissue architecture observed in the combined group resembled that of nitrofurazone, indicating *VOC*’s capacity to promote granulation tissue formation and resolution of inflammation, which are critical phases of repair [22].

Hydroxyproline analysis confirmed *VOC*’s ability to promote collagen deposition, a hallmark of wound healing. Elevated hydroxyproline levels suggest enhanced fibroblast activity and collagen biosynthesis, both essential for wound closure and mechanical strength [8]. Importantly, *VOC* demonstrated strong hyaluronidase inhibition, which may preserve hyaluronic acid and ECM integrity, thereby maintaining tissue hydration and support regeneration [23]. While hyaluronidase inhibition can preserve hyaluronic acid-rich ECM and hydration, some models show low-dose hyaluronidase can paradoxically stimulate hyaluronic acide synthesis and aid healing, indicating dose- and time-dependent outcomes [24,25]. Although *VOC* exhibited limited effects against collagenase and elastase, its selective hyaluronidase inhibition still represents a beneficial mechanism in maintaining ECM stability during healing. Excessive MMP-9 activity is a hallmark of impaired wounds and can hinder re-epithelialization; thus, selective modulation rather than broad suppression may be advantageous [26,27].

This selective enzyme inhibition pattern may have important physiological implications. Hyaluronidase inhibition helps preserve hyaluronic acid, a key glycosaminoglycan responsible for maintaining ECM hydration, viscoelasticity, and facilitating fibroblast migration during tissue regeneration. Preservation of hyaluronic acid has been associated with improved wound closure and reduced inflammation [25]. Conversely, the mild inhibition of collagenase and elastase observed with VOC may be advantageous, as these enzymes are necessary for normal matrix turnover and re-epithelialization. Excessive or non-selective inhibition could impair granulation tissue formation and delay remodeling. Hence, the selective modulation observed with VOC may favor a balanced wound-healing process—preserving ECM stability while allowing controlled proteolytic activity required for proper tissue repair [26,27].

In the present study, *VOC* treatment markedly increased the expression of VEGF and TGF-β1 in wound tissues, suggesting a dual mechanism involving both angiogenesis and ECM remodeling. VEGF is a key angiogenic mediator that promotes neovascularization and oxygen delivery to regenerating tissue [28,29], while TGF-β1 regulates fibroblast activation, collagen synthesis, and granulation tissue formation [30,31]. The simultaneous upregulation of these growth factors is consistent with the accelerated wound closure and increased tensile strength observed in *VOC*-treated groups. Previous studies have demonstrated that carvacrol, linalool, and eugenol—all major constituents of *VOC*—are capable of modulating angiogenic and fibrogenic pathways. Carvacrol has been reported to enhance VEGF-mediated angiogenesis [32], while eugenol stimulates fibroblast proliferation and TGF-β1 expression [33]. Linalool, on the other hand, exhibits anti-inflammatory and wound-healing-promoting effects Via downregulation of NF-κB and modulation of growth factors [13]. Together, these findings support the hypothesis that the synergistic interaction among *VOC* constituents contributes to the observed molecular and functional improvements.

While the overall results indicate a synergistic enhancement of wound-healing activity among *VOC* constituents, the possibility of antagonistic or non-additive interactions should also be considered. In complex essential oil mixtures, certain constituents may compete for the same molecular targets, alter membrane permeability, or affect compound stability, potentially reducing overall efficacy [18]. Such interactions are often concentration-dependent and influenced by the physicochemical compatibility of individual oils. Therefore, further fractionation and combination studies are warranted to determine the optimal ratios of bioactive components and to minimize potential antagonistic effects.

The oral dose of 40 mg/kg was selected considering the reported safety margins of carvacrol-, cineole-, and linalool-containing essential oils [12,13]. Previous in vivo studies demonstrated that such terpenoid-rich formulations are well tolerated up to 100 mg/kg without observable toxicity in rodents. In the current study, no behavioral or physiological abnormalities were detected, supporting the safety of the selected dosage.

Despite these promising results, certain limitations exist. The findings are based on animal models, which may not fully replicate human physiology. Furthermore, while enzyme inhibition assays provide mechanistic insights, additional molecular and signaling pathway analyses are required to clarify *VOC*’s effects on metalloproteinase regulation. Another limitation is that the present study did not include quantitative toxicological data such as body weight or serum biochemical parameters. Although no behavioral or physiological abnormalities were observed, future studies should include detailed systemic safety evaluations to substantiate the non-toxic nature of *VOC*. Ultimately, clinical validation will be essential before VOC can be recommended as a therapeutic agent for wound management.

## 4. Materials and Methods

### 4.1. Material

*VOC* was provided from Carmed İlaç Denizcilik Hayvancılık Sanayi ve Ticaret A.Ş. (Istanbul, Türkiye) in June 2025. *VOC* is formulated as a phytotherapeutic product composed of a mixture of essential oils derived from medicinal plants. The preparation includes oregano (*Origanum onites* L.), eucalyptus (*Eucalyptus globulus* Labill.), rosemary (*Rosmarinus officinalis* L.), sage (*Salvia officinalis* L.), common juniper (*Juniperus communis* L.) essential oils. All essential oils were verified for purity (>98%) by GC–MS prior to formulation. Olive oil (pharmaceutical grade, cold-pressed, Sigma-Aldrich, St. Louis, MO, USA) was used as the carrier for essential oil dilution and topical formulation. All other chemicals and reagents used in the study were of analytical grade.

### 4.2. GC-MS Analysis

The GC–MS analysis of *VOC* was carried out on a Thermo Scientific TSQ GC–MS system (Thermo Fisher Scientific, Waltham, MA, USA) equipped with a split/splitless injector and an electron ionization (EI) source operating at 70 eV. Separation was achieved using a capillary column (TG-5MS, 30 m × 0.25 mm i.d., 0.25 µm film thickness; Thermo Fisher Scientific, Waltham, MA, USA). The carrier gas was helium (99.999% purity), at a constant flow rate of 1.0 mL/min.

The injector temperature was set at 250 °C, and the injection volume was 1 µL of *VOC* diluted in *n*-hexane (1:100 *v*/*v*). The injection was performed in split mode (split ratio 20:1). The oven temperature program was as follows: initial temperature 60 °C (held for 2 min), ramped at 5 °C/min to 250 °C, then at 3 °C/min to 280 °C (held for 10 min). The transfer line and ion source temperatures were maintained at 280 °C and 230 °C, respectively.

Each sample of VOC was injected three times (*n* = 3) under identical chromatographic conditions to verify reproducibility. The retention times and relative peak areas of the major constituents showed a relative standard deviation (RSD) below 2%, confirming analytical consistency.

The system operated in full-scan mode (*m*/*z* 40–500). Peak identification was based on comparison of the obtained mass spectra with the NIST 14 spectral library and verified using authentic standards where available. The method reproducibility was confirmed by triplicate injections under identical chromatographic conditions.

Compound identification was performed by comparing the acquired mass spectra with entries in the NIST 14 mass spectral library (similarity index ≥ 90%) and by calculating linear retention indices (LRIs) using a homologous series of *n*-alkanes (C_8_–C_20_) analyzed under identical chromatographic conditions.

The LRIs were computed according to the Van Den Dool and Kratz method for temperature-programmed gas chromatography [34], using the following equation:

$$RI_{x}=100\times [n+\frac{t_{x}-t_{n}}{t_{n+1}-t_{n}}]$$

where $RI_{x}$ is the retention index of compound *x*, $t_{x}$ is the retention time of the compound, and $t_{n}$ and $t_{n+1}$ are the retention times of the *n*-alkanes eluting immediately before and after compound *x*, respectively.

The calculated LRIs were compared with published literature values to confirm compound identity. Only peaks with both spectral and LRI agreement (ΔRI < 10 units) were accepted as positively identified.

For further confirmation, the obtained mass-spectral data and calculated linear retention indices were compared with those reported in Adams (2007) [35]. Agreement between experimental and reference retention indices within ±10 units, together with matching fragmentation patterns, was considered sufficient for definitive compound identification.

### 4.3. In Vivo Experimental Studies

#### 4.3.1. Ethical Approval

The experimental protocol designed to evaluate the in vivo wound-healing effects of the *VOC* was reviewed and approved by the NESA Experimental Animals Local Ethics Committee (Decision no. 099). All experiments were conducted in accordance with the Guide for the Care and Use of Laboratory Animals.

#### 4.3.2. Experimental Animals

Male Sprague–Dawley rats (160–180 g) and Balb-C mice (25–30 g) were obtained from the NESA Laboratory Animal Research Facility. Animals were acclimatized for at least 3 days prior to the experiments under controlled conditions (22 ± 2 °C, 12 h light/dark cycle, relative humidity 55–60%). Standard pellet diet and water were provided ad libitum. Each experimental group consisted of seven animals. Small rodent models such as rats and mice are widely used to assess both functional and histological outcomes of wound healing [36,37].

#### 4.3.3. Animal Treatment and Experimental Groups

The animals were randomly allocated into experimental groups and treated orally, topically, or with a combination of both regimens. In the oral treatment group, *VOC* was administered at a dose of 40 mg/kg body weight, suspended in olive oil (0.2 mL), and delivered by gastric gavage twice daily (morning and evening). In the topical treatment group, *VOC* was prepared in olive oil and applied as a liquid formulation twice daily at a volume of 200 µL, corresponding to approximately X µL/cm^2^ of wound surface area, for 10 consecutive days. The combined treatment group received both oral and topical administrations concurrently. Control groups included a vehicle control (olive oil, 0.2 mL orally, twice daily), a negative control (no treatment), and a reference group treated with 0.2% nitrofurazone ointment applied topically twice daily. To prevent the animals from licking or ingesting the formulation, a restrainer was used briefly after each application.

The animals were randomly assigned to experimental groups using a computer-generated randomization list to minimize allocation bias. To prevent wound infection, the wound area was cleaned daily with sterile saline under aseptic conditions, and animals were housed individually in clean cages with autoclaved bedding to avoid cross-contamination. All procedures were performed under veterinary supervision and in full compliance with institutional ethical standards.

The oral dose of 40 mg/kg *VOC* was selected based on preliminary tolerability screening and reference data on the safety of essential oils containing carvacrol, 1,8-cineole, and linalool, which are major constituents of oregano, eucalyptus, and rosemary oils, respectively. Previous animal studies reported no adverse effects at oral doses up to 100 mg/kg for these essential oil components, while lower doses (<20 mg/kg) showed reduced pharmacological activity [12,13]. Therefore, a moderate dose of 40 mg/kg was adopted to balance efficacy and safety in the present experimental design.

#### 4.3.4. Linear Incision Wound Model

The linear incision model was established according to the method of Morton and Malone with modifications [38]. Rats were anesthetized with Ketamine (50 mg/kg, i.p.) and Xylazine (10 mg/kg, i.p.). The dorsal fur was shaved and disinfected with 70% ethanol. Two 5 cm long paravertebral full-thickness incisions were made 2 cm lateral to the midline using a sterile scalpel. Wound edges were closed with three interrupted sutures using sterile silk. Treatments were administered twice daily for 10 consecutive days. On day 11, sutures were removed, and animals were sacrificed under deep anesthesia. Wound tissue was excised with 2 cm margins. One specimen was processed for histology; the other was used to measure tensile strength with the continuous water flow technique, which determines wound breaking strength (g) by recording the hydrostatic pressure required to disrupt the healed incision [39].

$$\mathrm{Tensile}\;\mathrm{strength}\;\%=\frac{T_{\mathrm{sample}}-T_{\mathrm{control}}}{T_{\mathrm{control}}}\times 100$$

*T*_control_: The mean of tensile strength in the control group.

*T*_sample_: The mean of tensile strength in the test sample.

#### 4.3.5. Circular Excision Wound Model

This method is widely accepted as a reliable approach for monitoring epithelialization and contraction in experimental wound models [38].

The excision wound model was performed following the principles of Morton and Malone [38]. Mice were anesthetized with ketamine (50 mg/kg, i.p.) and xylazine (10 mg/kg, i.p.). After shaving and disinfecting the dorsal thoracic region, a circular full-thickness wound (314 mm^2^) was created using scissors and forceps. Day 0 was considered the day of surgery. Treatments were applied twice daily for 10 days. Wound contraction was monitored every two days by tracing wound margins on transparent acetate sheets, and wound areas were calculated planimetrically. The percentage of wound contraction was determined using the formula:

$$\mathrm{Wound}\;\mathrm{Contraction}\;\%=\frac{C_{\mathrm{control}}-C_{\mathrm{sample}}}{C_{\mathrm{control}}}\times 100$$

*C*_control_: The mean wound area of the control group.

*C*_sample_: The mean wound area of the test sample.

#### 4.3.6. Histopathological Examination

On day 11, wound tissues were harvested and fixed in 10% neutral buffered formalin for 48 h. Routine histological processing was performed, including dehydration in an ascending ethanol series (50–96%), clearing in xylene, and embedding in paraffin. Sections of 5 µm thickness were cut, mounted on normal and lysine-coated slides, and stained with hematoxylin–eosin (H&E). Sections were examined using a Nikon Eclipse Ci light microscope (Nikon Corporation, Tokyo, Japan) equipped with a KameraM^®^ digital analysis system (KameraM Software, Istanbul, Türkiye). Histological parameters evaluated included re-epithelialization, collagen deposition, granulation tissue formation, inflammation, and necrosis [40].

#### 4.3.7. Hydroxyproline Assay

Hydroxyproline determination remains the gold standard for collagen quantification in wound healing. Collagen content was assessed by determining hydroxyproline levels according to the modified method of Woessner [41]. Hydroxyproline standards were prepared by dissolving 5 mg hydroxyproline in 50 mL 0.001 N HCl and diluting to obtain concentrations of 0.5–2.5 µg/mL. Dried wound tissues were weighed, hydrolyzed with 5 mL 6 N HCl at 130 °C for 3 h, and neutralized to pH 6–7 with 2.5 N NaOH after addition of 0.02% methyl red indicator. Aliquots (2 mL) of samples and standards were oxidized with 1 mL freshly prepared chloramine-T at room temperature for 20 min. Subsequently, 1 mL perchloric acid and 1 mL Ehrlich’s reagent (*p*-dimethylaminobenzaldehyde) were added. After incubation in a water bath at 60 °C for 20 min and cooling for 5 min, absorbance was measured at 557 nm. Hydroxyproline content was expressed as µg/mg tissue.

### 4.4. In Vitro Enzyme Inhibition Assays

The inhibitory effects of *VOC* on extracellular matrix-degrading enzymes were assessed, as excessive activity of hyaluronidase, collagenase, and elastase contributes to impaired wound healing [6].

#### 4.4.1. Hyaluronidase Inhibition Assay

The assay was performed following Lee and Choi [42]. Fifty microliters of bovine testicular hyaluronidase (7900 U/mL) prepared in 0.1 M acetate buffer (pH 3.6) was mixed with 50 µL of *VOC* dissolved in 5% DMSO. Controls contained DMSO alone. After incubation at 37 °C for 20 min, 50 µL CaCl_2_ (12.5 mM) was added, followed by another 20 min incubation. Then, 250 µL sodium hyaluronate (1.2 mg/mL) was added, and the mixture was incubated at 37 °C for 40 min. The reaction was stopped by adding 50 µL 0.4 M NaOH and 100 µL 0.2 M sodium borate, followed by heating in boiling water for 3 min. After cooling, 1.5 mL *p*-dimethylaminobenzaldehyde reagent was added, and the mixture was incubated at 37 °C for 20 min. Absorbance was measured at 585 nm.

#### 4.4.2. Collagenase Inhibition Assay

The assay was carried out using *Clostridium histolyticum* collagenase according to Barrantes and Guinea [43]. The enzyme (0.8 U/mL) was prepared in 50 mM Tris buffer containing 10 mM CaCl_2_ and 400 mM NaCl. FALGPA (2 mM) was used as the substrate. Each reaction contained 25 µL buffer, 25 µL *VOC* sample, and 25 µL enzyme solution, incubated for 15 min at room temperature, followed by the addition of 50 µL substrate. Absorbance changes were recorded at 340 nm. Each experiment was performed in triplicate.

#### 4.4.3. Elastase Inhibition Assay

The elastase assay followed the method of Melzig et al. [44]. Human neutrophil elastase (17 mU/mL) was incubated with *VOC* in 0.1 M Tris-HCl buffer (pH 7.5) at 25 °C for 5 min. Subsequently, the synthetic substrate MAAPVN (500 µM) was added, and the mixture was incubated at 37 °C for 1 h. After incubation, 1 mg/mL soybean trypsin inhibitor was added, and the release of *p*-nitroaniline was measured at 405 nm.

Enzyme inhibition percentages were calculated as:

$$\%\;\mathrm{Inhibition}=\frac{A_{\mathrm{control}}-A_{\mathrm{sample}}}{A_{\mathrm{control}}}\times 100$$

*A*_control_: The absorbance of the control group.

*A*_sample_: The absorbance of the test sample.

#### 4.4.4. ELISA-Based Measurement of Wound Growth Factor (VEGF and TGF-β1) Responses

Growth factors were quantified using ELISA according to the manufacturers’ instructions. Mouse VEGF was measured using the Mouse VEGF DuoSet ELISA (R&D Systems/Bio-Techne, Minneapolis, MN, USA, DY493-05). TGF-β1 was measured using the Mouse TGF-β1 DuoSet ELISA (R&D Systems/Bio-Techne, Minneapolis, MN, USA, DY1679-05).

For TGF-β1, samples were acid-activated and neutralized before assay to convert latent TGF-β1 to its immunoreactive form, as recommended by the manufacturer. Plates were read at 450 nm with 540/570 nm wavelength correction, and concentrations were calculated from a four-parameter logistic (4-PL) standard curve.

Ancillary Reagent Kits 1 or 2 (R&D Systems) were used for plate coating, blocking, wash buffers, and TMB substrate according to the DuoSet protocol.

### 4.5. Statistical Analysis

All data were expressed as mean ± standard error of the mean (SEM). Differences between groups were analyzed by one-way analysis of variance (ANOVA), followed by the Student–Newman–Keuls Post Hoc test. A *p*-value < 0.05 was considered statistically significant. Statistical analyses were performed using Instat software (version 3.10, GraphPad Software, San Diego, CA, USA).

## 5. Conclusions

This study demonstrated that *VOC* possesses significant wound-healing activity through multifaceted mechanisms, including the enhancement of tensile strength, the acceleration of wound contraction, the promotion of re-epithelialization, increased collagen deposition, and selective hyaluronidase inhibition. Its phytochemical composition, dominated by carvacrol, along with other bioactive constituents such as thymol, 1,8-cineole, linalool, and eugenol, provides a strong basis for its pharmacological effects.

Taken together, these results highlight *VOC* as a promising complementary therapeutic product for wound care. However, further studies are warranted to isolate and quantify its active constituents, elucidate its molecular mechanisms, and rigorously assess its safety and clinical efficacy in human trials. Such investigations will be essential for translating *VOC* from experimental evidence into a standardized phytotherapeutic option for wound management.

## 6. Limitations and Clinical Translation

The present study demonstrated significant wound healing activity of *VOC* in rodent models; however, several limitations must be acknowledged. First, results obtained in animal models may not fully translate to human physiology, and interspecies differences could affect the clinical applicability of the findings. Second, while in vitro assays provided mechanistic insights into enzyme inhibition, molecular pathways underlying *VOC*’s effects were not extensively explored. Third, the study did not include comprehensive toxicological or pharmacokinetic evaluations, which are essential for establishing safety profiles. Future investigations should focus on controlled clinical trials in humans, dose optimization, long-term safety assessments, and mechanistic studies at the molecular level to support the potential translation of *VOC* into clinical wound management.

## Figures and Tables

**Table 1:** GC–MS analysis of the Vacrol Oil Combination.

RT (min)	Compounds	Concentration (%, *w*/*w*)
4.30	α-Pinene	3.5 ± 0.1
5.30	Camphene	0.4 ± 0.1
6.41	β-Pinene	0.7 ± 0.1
7.65	Δ-3-Carene	0.4 ± 0.1
8.21	Myrcene	0.3 ± 0.1
8.59	α-Terpinene	0.3 ± 0.1
9.17	Limonene	0.8 ± 0.1
9.42	1,8-Cineole	9.7 ± 0.1
10.64	δ-Terpinene	1.1 ± 0.1
11.38	*p*-Cymene	3.2 ± 0.1
15.59	α-Thujone	1.3 ± 0.1
16.11	β-Thujone	0.4 ± 0.1
16.68	Menthone	0.8 ± 0.1
18.03	Camphor	1.2 ± 0.1
19.06	Linalool	6.3 ± 0.1
20.07	Caryophyllene	0.9 ± 0.1
20.31	4-Terpineol	1.3 ± 0.1
21.22	Menthol	1.9 ± 0.1
21.76	Humulene	0.3 ± 0.1
22.44	Terpinenyl acetate	0.8 ± 0.1
22.55	Fencyl alcohol	0.6 ± 0.1
22.65	Borneol	0.9 ± 0.1
23.14	Bisabolene	1.1 ± 0.1
29.71	Cinnamaldehyde	4.3 ± 0.1
32.17	Eugenol	4.7 ± 0.1
32.60	Thymol	2.1 ± 0.1
33.16	Carvacrol	50.1 ± 0.1
51.10	Hexadecadienoic acid, methyl ester	0.6 ± 0.1

Retention times (RT), identified compounds, and their concentrations (% *w*/*w*) detected in the *VOC* by GC–MS analysis are presented.

**Table 2:** Effect of *VOC* on tensile strength in the linear incision wound model after 10 days of treatment. Data are expressed as mean ± SEM (*n* = 7).

Experimental Group	Wound Tensile Strength (g) ± S.E.M. (Wound Tensile Strength %)
Negative Control	101.08 ± 4.15
Vehicle (Olive oil)	140.13 ± 6.82 (38.6)
*VOC* (Oral)	171.58 ± 7.13 (22.4)
*VOC* (Topical)	205.21 ± 3.56 (46.4) *
*VOC* (Oral & Topical)	244.91 ± 3.14 (74.8) ***
Reference drug (Nitrofurazone)	235.04 ± 3.11 (67.7) ***

*: *p* < 0.05; ***: *p* < 0.001; SEM: Standard Error of the Mean. Statistical significance was determined by one-way ANOVA followed by Tukey’s post hoc test. Percentage wound tensile strength: The vehicle control group was compared to the negative control group. Test samples were compared to the vehicle control group.

**Table 3:** Percentage of wound contraction in the circular excision wound model during a 10-day treatment period. Data are expressed as mean ± SEM (n = 7).

Experimental Group	Wound Area ± SEM (Contraction %)					
	0	2	4	6	8	10
Negative Control	291.23 ± 4.73	295.75 ± 4.19	274.26 ± 3.98	242.51 ± 3.14	191.05 ± 3.11	105.27 ± 3.62
Vehicle (Olive oil)	295.83 ± 5.25	294.41 ± 5.17	255.07 ± 4.11 (6.9)	210.73 ± 3.57 (13.1)	164.19 ± 3.05 (14.1)	75.38 ± 2.61 (28.4)
*VOC* (Oral)	300.46 ± 4.17	290.16 ± 4.49	215.22 ± 4.03 (15.6)	154.26 ± 3.91 (26.8)	121.08 ± 3.53 (26.3)	54.76 ± 2.94 (27.4)
*VOC* (Topical)	293.17 ± 5.94	299.53 ± 4.34	206.36 ± 3.75 (19.1)	112.14 ± 3.01 (46.8) *	94.22 ± 3.01 (42.6) *	27.51 ± 2.64 (63.5) **
*VOC* (Oral & Topical)	298.39 ± 3.39	235.34 ± 3.08 (20.1)	171.15 ± 3.16 (32.9) *	97.04 ± 2.92 (53.9) **	56.09 ± 2.71 (65.8) **	00.00 ± 0.00 (100) ***
Reference drug (Nitrofurazone)	297.68 ± 4.86	276.33 ± 3.72 (6.1)	156.29 ± 3.82 (38.7) **	71.54 ± 3.28 (66.1) **	42.15 ± 2.43 (74.3) **	14.70 ± 2.08 (80.5) **

*: *p* < 0.05; **: *p* < 0.01; ***: *p* < 0.001; SEM: Standard Error of the Mean. Statistical significance was determined by one-way ANOVA followed by Tukey’s post hoc test. The vehicle control group was compared to the negative control group. Test samples were compared to the vehicle control group.

**Table 4:** Effect of *VOC* on hydroxyproline levels (µg/mg tissue) in wound tissues, reflecting collagen content. Data are expressed as mean ± SEM (*n* = 7).

Material	Hydroxyproline Content (µg/mg Tissue) ± S.E.M.
Negative Control	7.65 ± 1.81
Vehicle (Olive oil)	12.57 ± 2.09
*VOC* (Oral)	19.72 ± 1.63
*VOC* (Topical)	22.34 ± 1.08 *
*VOC* (Oral & Topical)	32.06 ± 1.57 **
Reference drug (Nitrofurazone)	49.51 ± 1.13 ***

*: *p* < 0.05; **: *p* < 0.01; ***: *p* < 0.001; SEM: Standard Error of the Mean. Statistical significance was determined by one-way ANOVA followed by Tukey’s Post Hoc test. The vehicle control group was compared to the negative control group. Test samples were compared to the vehicle control group.

**Table 5:** In vitro enzyme inhibition activity of *VOC* against hyaluronidase, collagenase, and elastase. Data are expressed as mean ± SEM.

Material	Concentration (µg/mL)	Hyaluronidase Inhibition (%) ± SEM	Collagenase Inhibition (%) ± SEM	Elastase Inhibition (%) ± SEM
*VOC*	100	71.63 ± 0.52 ***	9.84 ± 1.53	8.26 ± 1.19
Tannic acid	100	83.17 ± 0.34 ***	-	-
Epigallocatechin gallate	100	-	49.52 ± 0.98 **	67.11 ± 0.92 ***

** p < 0.01; ***: *p* < 0.001; SEM: Standard Error of the Mean.

## Data Availability

The dataset used and/or analyzed during the current study available from the corresponding author on reasonable request.

## Abbreviations

The following abbreviations are used in this manuscript:

ANOVA	Analysis of Variance
CaCl_2_	Calcium chloride
CRM	Certified Reference Material
DMSO	Dimethyl sulfoxide
ECM	Extracellular matrix
ELISA	Enzyme Linked Immunosorbent Assay
FALGPA	N-(3-[2-furyl]acryloyl)-Leu-Gly-Pro-Ala
GC-MS	Gas Chromatography–Mass Spectrometry
H&E	Hematoxylin-Eosin
HCl	Hydrochloric acid
HRP	Horseradish peroxidase
i.p.	Intraperitoneal
MAAPV	N-Methoxysuccinyl-Ala-Ala-Pro-Val-p-nitroanilide
NaCl	Sodium chloride
NaOH	Sodium hydroxide
NIST	National Institute of Standards and Technology
RT	Retention time
SEM	Standard Error of the Mean
TGF- β1	Transforming growth factor beta 1
TMB	3,3′,5,5′-Tetramethylbenzidine
TÜBİTAK	Türkiye Bilimsel ve Teknolojik Araştırma Kurumu
VEGF	Vascular endothelial growth factor
VOC	Vacrol Oil Combination
*w*/*w*	Weigh/weight
